# Novel compound heterozygous mutations of the *FBP1* gene in a patient with hypoglycemia and lactic acidosis: A case report

**DOI:** 10.1002/mgg3.2339

**Published:** 2023-12-19

**Authors:** Bin Xin, Haiming Chen, Tianyi Liu, Yue Wu, Qingyang Hu, Xue Dong, Zhong Li

**Affiliations:** ^1^ Department of Pharmaceutics Dalian Women and Children's Medical Group Dalian Liaoning China; ^2^ College of Pharmacy Dalian Medical University Dalian Liaoning China; ^3^ Department of Emergency Medicine Dalian Women and Children's Medical Group Dalian Liaoning China

**Keywords:** *FBP1* gene, fructose‐1,6‐bisphosphatase (FBPase) deficiency, hypoglycemia, mutation

## Abstract

**Background:**

Fructose‐1,6‐bisphosphatase (FBPase) deficiency, caused by an *FBP1* mutation, is an autosomal recessively inherited metabolic disorder characterized by impaired gluconeogenesis. Due to the rarity of FBPase deficiency, the mechanism by which the mutations cause enzyme activity loss still remains unclear.

**Methods:**

We report a pediatric patient with typical FBPase deficiency who presented with hypoglycemia, hyperlactatemia, metabolic acidosis, and hyperuricemia. Whole‐exome sequencing was used to search for pathogenic genes, Sanger sequencing was used for verification, and molecular dynamic simulation was used to evaluate how the novel mutation affects FBPase activity and structural stability.

**Results:**

Direct and allele‐specific sequence analysis of the *FBP1* gene (NM_000507) revealed that the proband had a compound heterozygote for the c. 490 (exon 4) G>A (p. G164S) and c. 861 (exon 7) C>A (p. Y287X, 52), which he inherited from his carrier parents. His father and mother had heterozygous G164S and Y287X mutations, respectively, without any symptoms of hypoglycemia.

**Conclusion:**

Our results broaden the known mutational spectrum and possible clinical phenotype of *FBP1*.

## Graphical abstract

The case report describes the clinical syndromes of genetic testing of FBPase deficiency in a Chinese boy. It provides evidence that proband impaired FBPase activity may be due to this novel compound heterozygous mutations c. 490 (exon 4) G>A (p. G164S) and c. 861 (exon 7) C>A (p. Y287X, 52) of the FBP1 gene.

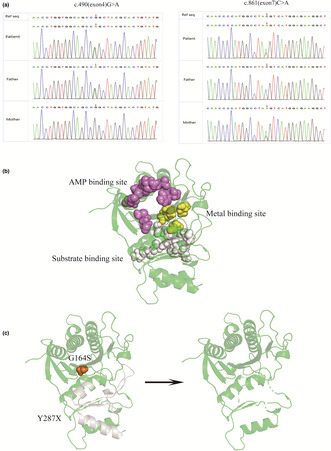

## 1 INTRODUCTION

Fructose‐1,6‐bisphosphate (FBPase) is a pivotal regulatory enzyme of gluconeogenesis that catalyzes the hydrolysis of fructose‐1,6‐bisphosphate to fructose‐6‐phosphate and inorganic phosphate (Rahil et al., 1982). Two isoforms of FBPase have been discovered in mammals encoded by two genes: a liver FBPase (*FBP1*), which is expressed primarily in the liver and kidney, and a muscle FBPase (*FBP2*), which is found exclusively in muscle tissue (Matsuura et al., 2002). These two enzymes exhibit around 77% similarity at the amino acid level and have almost complete identity in regions of the enzyme's active site. The *FBP1* gene consists of seven exons of more than 31 kb in length on chromosome 9q22.2‐q22.3 and six introns in the same position in the rat gene (El‐Maghrabi et al., 1995).

FBPase deficiency (OMIM:229700) was first described by Baker and Winegrad in 1970 (Baker & Winegrad, 1970), and Kikawa et al. subsequently identified three *FBP1* mutations associated with FBPase deficiency in 1997 (Kikawa et al., 1997). Defective FBPase enzymes block the conversion of substrates such as amino acids, lactate, glycerol and pyruvate to glucose, leading to hypoglycemia and the accumulation of gluconeogenic substrates (Åsberg et al., 2010; Baker & Winegrad, 1970). Infection, prolonged fasting, or massive fructose ingestion can trigger acute crises. Clinical manifestations of patients with FBPase deficiency include severe hypoglycemia, lactic acidosis, elevated liver enzymes, and metabolic acidosis. Patients in infancy and toddlerhood are prone to acute episodes of severe hypoglycemia and lactic acidosis that can lead to seizures, brain damage, and even life‐threatening (Kamate et al., 2014; Liang et al., 2023; Mei et al., 2019). FBPase deficiency is generally believed to be extremely rare with an estimated incidence between 1: 350,000 and <1: 900,000 in the Dutch and French populations, respectively (Lebigot et al., 2015; Visser et al., 2004), but it may be more common in populations with a higher rate of consanguinity. Here, we describe the case of a pediatric patient who was diagnosed with FBPase deficiency by genetic sequencing and exhibited compound heterozygous mutations of the *FBP1* gene.

## 2 MATERIALS AND METHODS

### 2.1 Ethical compliance

This study has been approved by the Medical Ethics Committee of the Dalian Women and Children's Medical Group.

### 2.2 Whole‐exome sequencing and Sanger sequencing

Previous research has demonstrated that genetic analyses using next‐generation sequencing are useful for the molecular diagnosis of complicated metabolic diseases, including FBPase deficiency (Li et al., 2017). Thus, we performed whole‐exome sequencing for samples from the proband and his parents to identify potential underlying genetic defects. Whole‐exome sequencing was performed using IDT The xGen Exome Research Panel v1.0 whole‐exome capture chip, sequenced by Illumina NovaSeq 6000 series sequencers. The coverage of the target sequence was at least 99%. The ABI3730 sequencer was used for Sanger sequencing verification, and the sequence analysis software obtained the verification results.

### 2.3 Molecular dynamic simulations

Molecular dynamic (MD) simulations were performed using the program GROMACS 2023‐1 (Van Der Spoel et al., 2005), with a crystal structure of *FBP1* (DOI: 10.2210/pdb5ZWK/pdb) downloaded in the RCSB PDB database (https://www.rcsb.org/). The Pymol 2.6.0 software was used to delete 52 untranslatable amino acids after tyrosine 287 of the *FBP1* gene to generate a model for the Y287X mutation. The Charmm36 force field was used for dynamic simulations. Both structures were solvated with a three‐point water model (SPC) in a cube box (solute box distance of 1.2 nm) under periodic boundary conditions. Starting structures were energy minimized until convergence at Fmax <1000 kJ/mol/nm. A 100 ps position‐restrained NVT equilibration simulation was run for water relaxation at 310 K using a modified Berendsen (velocity rescaling) thermostat, followed by a 100 ps NPT equilibration simulation with Berendsen pressure coupling. After equilibration, an unrestrained 100 ns molecular dynamics simulation was run. After the simulation, the “gmx trjconv” command was used to correct the trajectory of the protein movement.

## 3 RESULTS

### 3.1 Case report

The patient was a 7‐year‐old boy, born by cesarean section at 38 weeks gestation, the first child of healthy non‐consanguineous parents. His physical and psychomotor development was generally normal. The patient had three hospitalizations with episodic vomiting and two episodes of transient temperature elevation; biochemical tests: hypoglycemia, metabolic acidosis, elevated lactate, hyponatremia, two elevated triglycerides and one elevated uric acid (the uric acid test wasn't carried out at Dalian Women and Children's Medical Group; it was done at the other institution), interictal status was good. All of the clinical and laboratory data in the patient's three hospitalizations are presented in Table 1. After the diagnosis was confirmed by genetic testing, there were 6 hospitalizations between the ages of 4 and 7 years, with 4 episodes of “vomiting, hypoglycemia, metabolic acidosis and elevated lactic acid,” including 1 febrile hypoglycemic convulsion at the age of 5 years and 1 hypoglycemic convulsion at the age of 7 years, which presented as a grand mal seizure. Improvement after administration of glucose intravenous infusion and expectant treatment, with the average hospital stay was about 3 days. The patient's clinical manifestations were hypoglycemia, hyperlactatemia, metabolic acidosis and ketosis, and infection was the cause of the attack. After each admission, the patient was given glucose intravenous infusion, intravenous rehydration, correction of acidosis, anti‐infection, and other treatments to recover quickly.

**TABLE 1:** Summary of clinical manifestations of patients.

Patient	First hospitalization	Second hospitalization	Third admission
Age	1 year‐11 months	2 years‐4 months	2 years‐7 months
*T* (°C)	37.0	37.7	37.5
*Results of blood chemistry*			
WBC (5.1–14.1 × 10^9^/L)	26.83	16.50	17.02
NEUT (13–55%)	81.5	75.6	81.7
LYMPH (33–77%)	14.1	17.4	13.7
HGB (107‐141 g/L)	115	127	134
PLT (190–524 × 10^9^/L)	442	487	258
CRP (0–10 mg/L)	0.74	3.38	4.55
Lactic acid (0.7–2.1 mmol/L)	10.6	12.3	10.4
PH of arterial blood (7.35–7.45)	6.96	7.11	7.12
Blood glucose (3.9–6.1 mmol/L)	3.25	2.5	14.9^a^
Base excess (−3–3 mmol/L)	−23.7	−20.3	−21.5
Sodium (135–145 mmol/L)	127.10	129	124
Potassium (3.5–5.0 mmol/L)	5.06	3.7	5.0
Calcium (2.08–2.60 mmol/L)	0.27	1.28	1.22
Chloride (98–100 mmol/L)	71	–	–
Triglyceride (0.45–1.55 mmol/L)	–	1.86	4.74
Ketones in urine	–	+ + +	+ + +
Uric acid (146–369 μmol/L)	709.56 μmol/L	–	–

Abbreviations: CRP, C‐reactive protein; HGB, hemoglobin; LYMPH, lymphocytes; NEUT, neutrophils; PLT, blood platelet; T, body temperature; WBC, white blood cell count; −, the examination was not performed.

^a^ The blood glucose value after 200 mL intravenous infusion of potassium chloride and glucose.

### 3.2 Identification of the mutations in the *FBP1* gene

**FIGURE 1:**
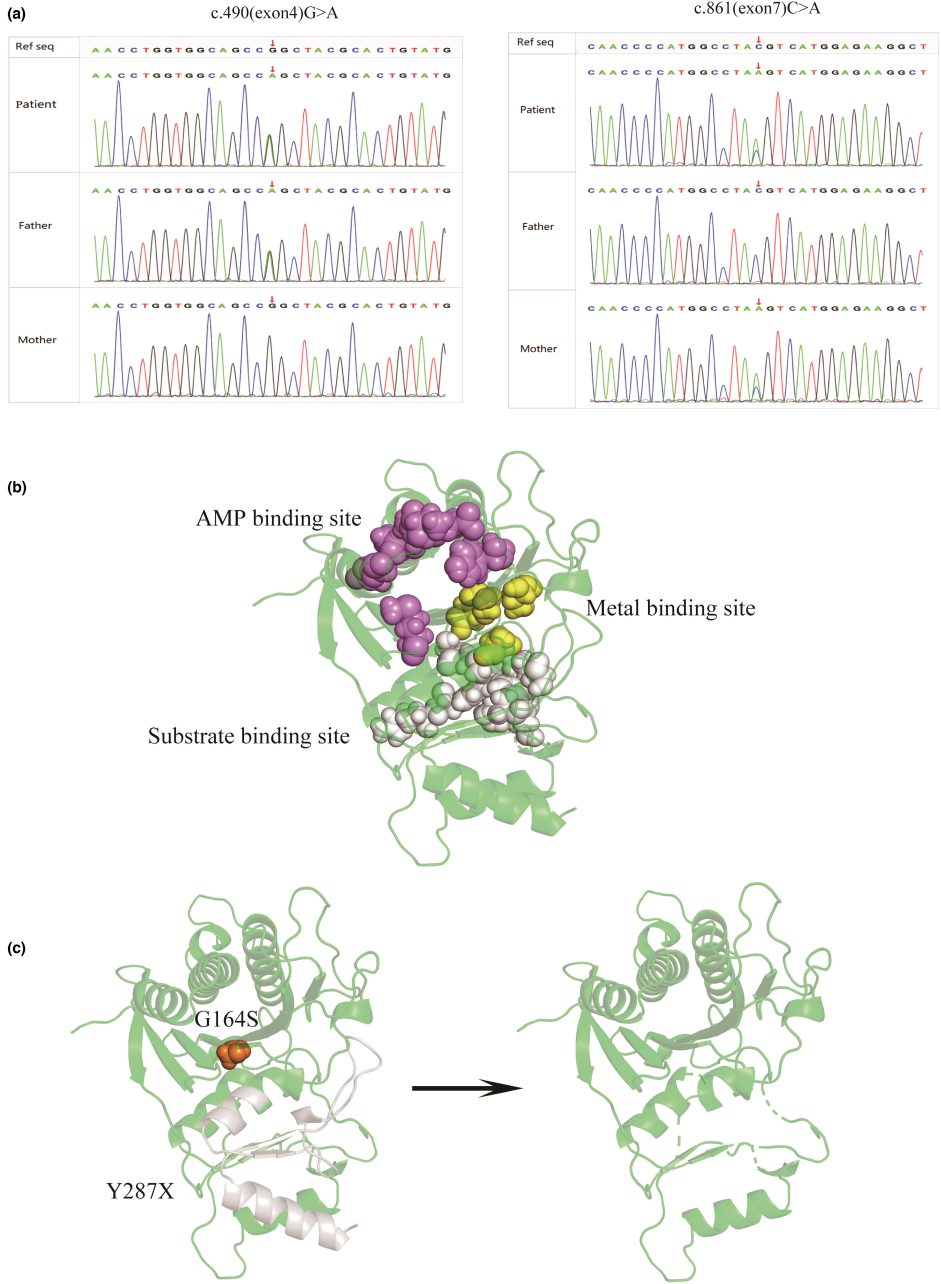
*FBP1* mutations were identified in a pediatric patient with hypoglycemic acidosis. (a) Compound heterozygous mutations in the *FBP1* gene, G164S and Y287X, were identified in the patient and validated using Sanger sequence analysis. (b) The structure of the *FBP1* based on the protein data bank (DOI: 10.2210/pdb1FBP/pdb) is shown. Purple sphere: AMP binding sites; yellow sphere: metal binding site; gray sphere: substrate‐binding site. (c) G164S and Y287X mutation sites are shown as orange spheres and gray cartoons, respectively.

Two heterozygous variants were identified in the *FBP1* gene (Figure 1a). One was a guanine missense mutation to adenine at base 490 in exon 4, where the amino acid changed from glycine to serine (c.490 G>A, p. Gly164Ser). This mutation was inherited from the patient's father. It was present in the NCBI dbSNP (rs121918188), the dbSNP database with a minor allele frequency of 0.000008, and ExAC databases with a minor allele frequency of 0.0001. The other one was a mutation from cytosine to adenine at base 861 of exon 7, where the amino acid 287 changed from tyrosine to a stop codon, which prevents all 52 amino acids after amino acid 287 from being translated, resulting in the protein being shorted by 52 amino acids (c.861 C>A, p. Tyr287Stop,52). This mutation, inherited from the patient's mother, was a novel variant not found in the dbSNP, 1000 Genomes Project, and Exome Variant Server databases. Sanger sequencing confirmed these variants were compound heterozygous and inherited from each parent.

The human liver FBPase consists of four identical polypeptide chains. Each of these chains contains 338 amino acid residues, which are assembled as relatively flat tetramers with subunits conventionally labeled C1–C4 (Kaur et al., 2017). These homotetramers consist of two intimate dimers. Figure 1b shows the structure of the *FBP1*. Fructose 2,6‐bisphosphate (F2,6BP) and AMP function synergistically as inhibitors to regulate the intracellular activity of the FBPase enzyme. F2,6BP binds at the substrate binding site of FBPase, hence a competitive inhibitor of FBPase, while AMP binds at the allosteric site, a non‐competitive inhibitor. FBPase catalyzes the hydrolysis of fructose 1,6‐bisphosphate to fructose 6‐phosphate in the presence of divalent cations, such as magnesium, manganese, or zinc (Kaur et al., 2017; Ke et al., 1990). We found that neither G164S nor Y287X, 52 corresponded to pivotal amino acid residues within functional motifs, such as AMP binding sites, substrate binding sites, or metal binding sites (Figure 1b). However, the 52 amino acids at the C‐terminal cannot be translated correctly, resulting in an incomplete polypeptide chain. The untranslatable sites are close to metal ions and substrate binding sites, affecting the binding of metal ions and substrate.

G164 is located in the β‐strand structure (Figure 1c). Previous studies have shown that the G164S mutation of the *FBP1* gene is expected to result in certain conformation modification or protein stability because the highly hydrophilic serine substitutes for the low‐hydrophilic glycine. The FBPase activity of purified protein from a mutant G164S cDNA construct, which was overexpressed in Escherichia coli, was found to be markedly decreased (Kikawa et al., 1997). Other studies have confirmed that the FBPase activity of the mutant G164S *FBP1* cDNA, which was overexpressed in COX‐7 cells, was significantly less than that of the wild type (wt) (Moon et al., 2011). We used dynamic simulation to investigate whether the novel mutation (Y287X, 52) affects FBPase activity and structural stability.

### 3.3 The results of dynamic simulation

**FIGURE 2:**
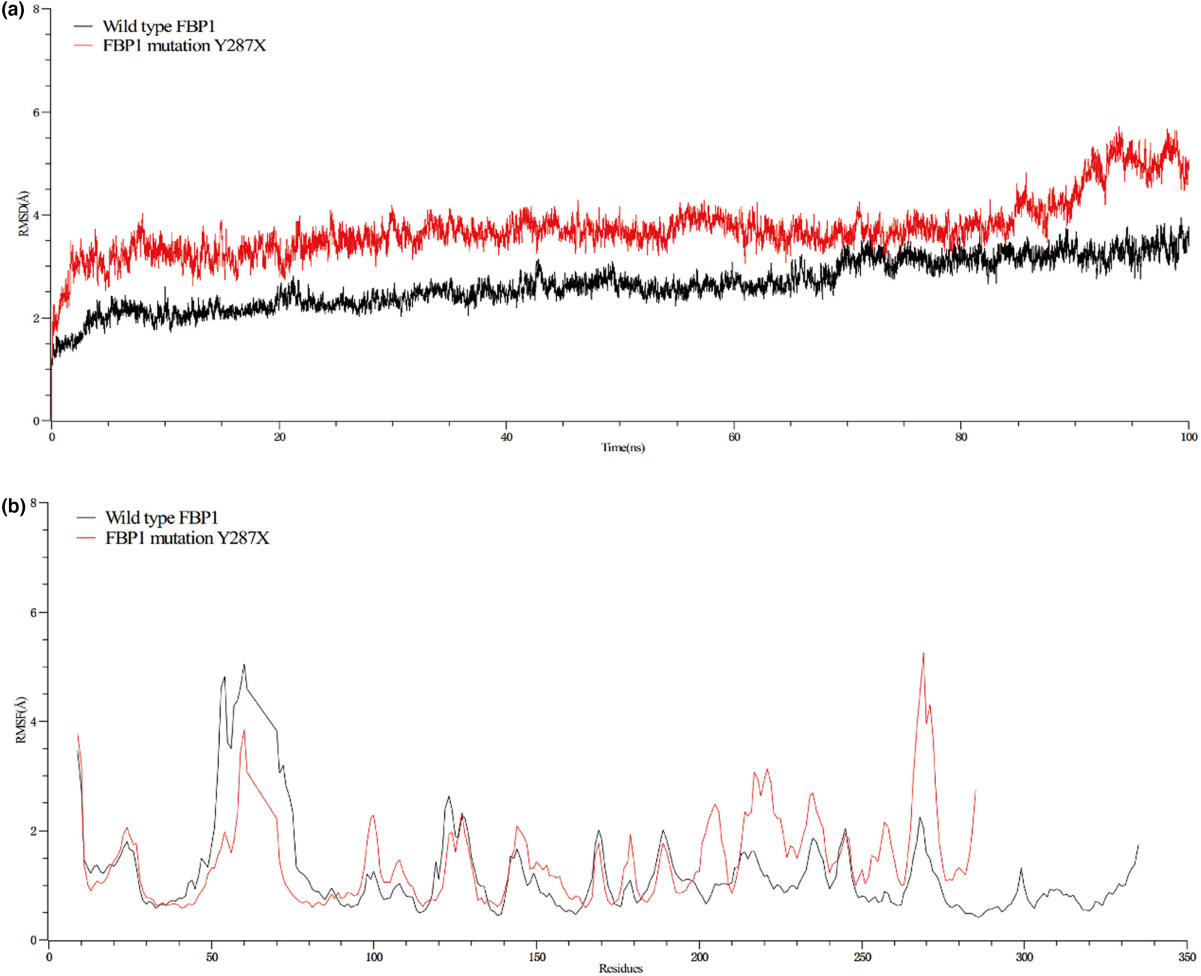
The dynamic simulation data of wild type *FBP1* and Y287X mutation. (a) MD Simulation data showing RMSD. (b) MD Simulation data showing RMSF.

We calculated the root‐mean‐square deviations (RMSD) of wild‐type *FBP1* and mutant Y287X's carbon skeleton to assess the system's convergence. The increasing all‐atoms backbone RMSD values for Y287X compared to the converging values for wt indicate a destabilizing effect. The backbone atoms of wt display minor fluctuation after 20 ns. Y287X, the RMSD increases gradually with a maximum deviation of 5.6 Å in the last 10 ns without further convergence (Figure 2a). The mean RMSD of the mutation Y287X (3.72) was larger than that of the wt RMSD (2.64). Compared with wild‐type *FBP1*, mutant Y287X significantly changed the protein structural topology. The root‐mean‐square fluctuation (RMSF) was used to further study the vibration amplitude of different residues in wild‐type *FBP1* and mutant Y287X to evaluate protein stability. The RMSF of Y287X shows notably increased fluctuation up to 5.2 Å compared to wt in the range of residues between 265 and 275(Figure 2b). However, the metal ion binding sites (P271) and substrate binding sites (Y264, K269, K274) are near residue 270, affecting the activity of the FBPase enzyme.

### 3.4 Treatment and follow‐up

The patient was given glucose intravenous infusion, intravenous rehydration, correction of acidosis, and anti‐infection therapy on three admissions, and his condition recovered quickly. After the diagnosis of FBPase deficiency by genetic testing, the child had two convulsions during the follow‐up period. Despite the parents knowing to pay attention to the diet and avoid prolonged fasting, control over diet and life was not satisfactory. Therefore, it is important to continuously strengthen late management to prevent hypoglycemia from causing convulsions. The patient was advised to avoid long‐term fasting, starvation and infection, increase feeding frequency, and limit the intake of high‐protein, high‐fat foods. In addition, sorbitol and sucrose should be avoided (chewing gums, high‐fructose corn syrup, iced tea, sports drinks, etc.). Fruits, especially apples, pears, grapes and cherries, contain a higher ratio of fructose to glucose. Glycerol solutions that contain 5% fructose are commonly used to treat brain edema but should be avoided in FBPase deficiency (Kamate et al., 2014). In case of fever or feeling down, administer glucose immediately orally or intravenously as a way. If necessary, continuous feeding by a gastric tube is required to maintain a stable blood glucose level during acute infection. Finally, the use of fructose‐free food and avoidance of prolonged fasting with the administration of uncooked corn starch (2 g/kg) mixed with water at midnight were of benefit to our patients, preventing nocturnal hypoglycemia and improving their clinical response to illness (Burlina et al., 1990). The overall prognosis for children with FBPase deficiency is good, with tolerance to starvation increasing with age (Åsberg et al., 2010).

## 4 DISCUSSION

In this study, we presented a patient with FBPase deficiency, including hypoglycemia, hyperlactatemia, metabolic acidosis and hyperuricemia. Exome sequencing and genetic data analysis revealed that phenotypic manifestations may be explained by a novel compound heterozygous mutation of the *FBP1* gene. A G164S and InsG960_961 heterozygote mutation has been characterized (Kikawa et al., 1997), but the G614S and Y287X heterozygote mutation is novel. Gene tracking showed that the proband's father had a heterozygous G164S mutation, and the mother had a heterozygous Y287X mutation. These results indicate that the proband inherited the G164S and Y287X mutation from each parent. Therefore, the FBPase deficiency of the proband was an autosomal recessive disorder at the molecular level. To date, 60 pathogenic variants have been reported in the *FBP1* gene worldwide, including missense variants, indels, splice variants, single exon deletions, and complete gene deletions (Liang et al., 2023).

FBPase deficiency is a severe disorder that can be fatal, mainly in newborns. FBPase deficiency can easily be confused with mitochondrial dysfunction and other metabolic disorders, such as glucose‐6‐phosphatase deficiency. There is a high rate of misdiagnosis of FBPase deficiency, especially without definitive biochemical indicators. More than 90% of children with FBPase deficiency had hypoglycemia and metabolic acidosis, and most had hyperlactatemia, but these biochemical changes are not specific. The diagnosis of FBPase deficiency was initially determined based on clinical symptoms and FBPase activity (Shin, 1993). The detection of glycerol, especially glycerol‐3‐phosphate, in the urine of children with hypoglycemia and acidosis may provide clues to the diagnosis of FBPase deficiency (Kato et al., 2015). FBPase activity was measured using liver biopsies or peripheral blood lymphocytes, but results were inconsistent, and the reliability of these methods remains controversial (Åsberg et al., 2010). Early and definitive diagnosis helps to improve patient quality of life and survival rate (Ergoren et al., 2020). The rapid development of gene sequencing technology has made genetic analysis an effective strategy to facilitate the diagnosis of rare genetic diseases. Children with FBPase deficiency can lead a normal life by avoiding fasting and appropriate intervention and monitoring during crises.

The c.490G>A mutation has been detected in Japanese and Korean patients. This mutation site is commonly found in reported cases in China, so it can be inferred that c.490G>A may be a hotspot mutation in Chinese or even Asian populations (Jian et al., 2018; Kikawa et al., 1997; Li et al., 2017; Lyu et al., 2019; Moon et al., 2011). The biochemical phenotypes of all previously reported *FBP1* missense mutations are classified into three functional categories. Type 1 mutations are located at pivotal residues in enzyme activity motifs and have no effects on protein expression. Type 2 mutations structurally cluster around the substrate binding pocket and are associated with reduced protein expression due to protein misfolding. Type 3 mutations are structurally distant from sites associated with the enzyme activity motif and substrate binding pocket. These mutations exhibited normal FBPase enzyme activity (Sakuma et al., 2023). The G164S belongs to the type 2 mutation because it will likely be located outside the important amino acid residues in the functional motif. It appears to cluster around the substrate binding pocket, in line with the changes in amino acid hydrophobicity. Type 2 mutations decreased protein expression and caused aggregation in the cytoplasm, possibly due to protein misfolding. The result shows that protein misfolding, particularly for Type 2 mutations, plays a critical role in driving the pathophysiology of FBPase deficiency. The G164S *FBP1* mutants exhibit decreased *FBP1* protein expression and a loss of FBPase enzyme activity.

A premature stop codon in the protein often results in nonsense‐mediated mRNA decay (Frischmeyer et al., 2002). To date, the reported nonsense mutations have Y216X, Q229X. Analysis of enzyme activity in monocytes stimulated by calcitriol showed no detectable FBPase level found in the monocytes of boys with the Y216X mutation (Åsberg et al., 2010). The Q229X mutation changed the amino acid codon from glutamine (CAG) in position 229 to a stop codon (TAG), which caused a shortening of the protein from the normal 338 amino acids to 228. The shortened protein lacks a major portion of the active site and is probably without enzymatic activity (Lebigot et al., 2015; Prahl et al., 2006). Consequently, amino acid residues at the C‐terminal seem to be essential for normal FBPase activity. The secondary or tertiary structure of the FBPase protein may be impacted by the deletion of amino acid residues at the C‐terminal by an unidentified mechanism (Kikawa et al., 1997). The Y287X mutation is the conversion of cytosine to adenine, which converts the translation tyrosine to the stop codon and prematurely terminates the polypeptide chain synthesis, resulting in an incomplete polypeptide chain. The dynamic simulation of wild‐type *FBP1* and Y287X mutant showed that the mutant with 52 amino acids missing was structurally unstable, and the binding sites of metal ions and positions were affected. The FBPase activity of the Y287X mutant was significantly decreased compared with that of the wild type, and the activity may even be lost. According to the standards issued by the American College of Medical Genetics and Genomics (Richards et al., 2015), the novel mutation of Y287X is pathogenic (PVS1 + PM2).

## 5 CONCLUSIONS

In summary, this pediatric patient with FBPase deficiency was described and diagnosed by trio whole‐exome testing, identifying 2 mutations of the *FBP1* gene, both located on chromosome 9. The first variant, c. 490 (exon 4) G>A (p. Gly164Ser), was inherited from the patient's father. The mutation 490G>A (p. Gly164Ser) was previously identified as pathogenic. The second variant, c. 861 (exon 7) C>A (p. Tyr287Stop, 52) from the patient's mother; this type had not previously been reported. Our study contributes to the mutation spectrum of *FBP1* by identifying a novel missense variant in the Chinese population.

## AUTHOR CONTRIBUTIONS

Zhong Li conceptualized and designed this study, analyzed the results, and was the author primarily responsible for drafting the article.Bin Xin performed experiments, interpreted the data and drafted the manuscript. Haiming Chen provided clinical evaluation and information. Tianyi Liu contributed to experiments. Yue Wu, Qingyang Hu, and Xue Dong performed data analysis.All authors read and approved the final manuscript.

## FUNDING INFORMATION

This work was supported by a grant from the Dalian Municipality Medical Technological Innovation Project (No. 2019J13SN84).

## CONFLICT OF INTEREST STATEMENT

The authors declare no conflict of interest.

## ETHICS STATEMENT

This study has been approved by the Medical Ethics Committee of the Dalian Women and Children's Medical Group. This article was a case report, and all treatment measures were necessary to save the patient's life without any research purpose. Therefore, our institutional ethics committee granted us a waiver of informed consent for publication.

## Data Availability

The data that support the findings of this study are available from the corresponding author upon reasonable request.
